# Determination of Chloride Content in Cementitious Materials: From Fundamental Aspects to Application of Ag/AgCl Chloride Sensors

**DOI:** 10.3390/s17112482

**Published:** 2017-10-29

**Authors:** Farhad Pargar, Dessi A. Koleva, Klaas van Breugel

**Affiliations:** Faculty of Civil Engineering and Geosciences, Department Materials and Environment, Delft University of Technology, Stevinweg 1, 2628 CN Delft, The Netherlands; d.a.koleva@tudelft.nl (D.A.K.); k.vanbreugel@tudelft.nl (K.v.B.)

**Keywords:** Ag/AgCl electrode, chloride sensor, concrete, cement, chloride

## Abstract

This paper reports on the advantages and drawbacks of available test methods for the determination of chloride content in cementitious materials in general, and the application of Ag/AgCl chloride sensors in particular. The main factors that affect the reliability of a chloride sensor are presented. The thermodynamic behaviour of silver in the presence or absence of chloride ions is described and kinetic restrictions are addressed. The parameters that can affect the activity of chloride ions in the medium and/or the rate of ion exchange and dissolution/precipitation processes at the sensor’s surface are also considered. In this regard, the contribution of morphology and microstructure of the AgCl layer, binding of chloride ions and the compactness of hydration products around the chloride sensor are highlighted. The important parameters for a reliable sensor’s response are discussed and the possible causes of inaccuracies are evaluated.

## 1. Introduction

Chloride ions are among the primary causes of steel corrosion in reinforced concrete structures. Determination of the chloride content in the concrete cover and near the steel reinforcement is needed for evaluating the risk of corrosion. The drawbacks of conventional techniques for determination of the chloride content call for novel and reliable techniques. One of the first documented attempts for non-destructive determination of the chloride content in cementitious materials was reported in the early 1990s, when Ag/AgCl chloride sensors were embedded in mortar specimens [1]. However, the literature on the performance of chloride sensors in cementitious materials is still scarce and the interpretation of sensors’ readings is far from straightforward.

**Figure 1:**
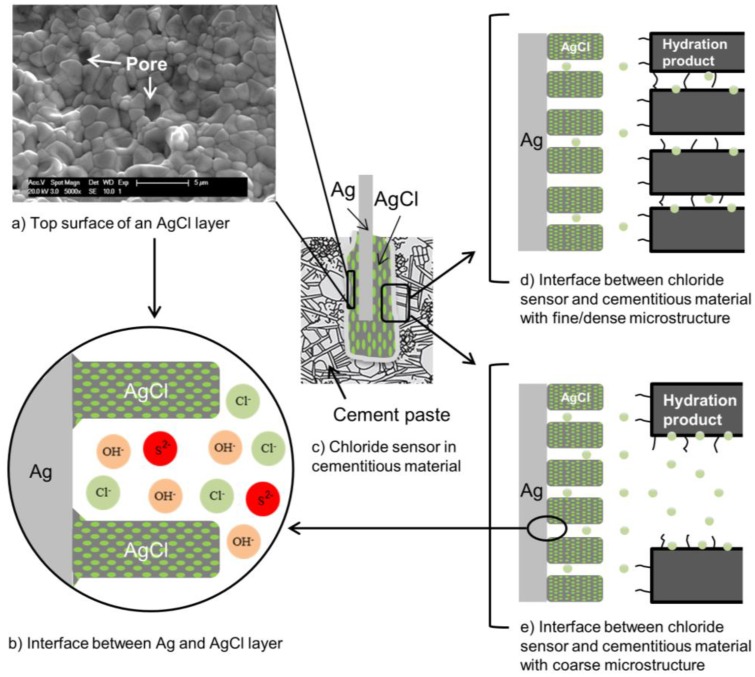
Schematic representation of chloride sensor embedded in a cementitious matrix.

The performance of embedded chloride sensor in cementitious materials depends on the physical condition of the interfaces (Ag/AgCl/cement paste) at the sensor’s surface as well as the pore solution composition. In this regard, a schematic presentation of Ag/AgCl sensor in cement paste is depicted in Figure 1. This figure illustrates some morphological and microstructural features of the AgCl layer and adjacent cementitious material, both important for the sensor’s response. The pores at the surface of the AgCl layer provide a pathway for penetration of ions into the layer (Figure 1a). The presence of different ions in the pore channels of this layer, subsequently affects the sensor’s response (Figure 1b). When the chloride sensor is embedded in cementitious materials (Figure 1c), the microstructure of these cementitious materials is also important for the electrochemical reactions relevant to the sensor’s response (Figure 1d,e). This is mainly in view of pore solution composition and compactness of hydration products at the interface between sensor and cementitious materials.

The open circuit potential (OCP) of a chloride sensor is a function of the surface characteristics of the sensor and the rate of electron/ion transfer at the sensor’s surface [2]. In this regard, the surrounding medium can affect the thermodynamically plausible reactions at the chloride sensor’s surface. For example, the binding of chloride ions to the hydration products, together with the relative distribution and compactness of hydration products around the sensor, make the chloride ions less “effective” in the pore solution than expected based on their concentration. Moreover, the sensitivity of a chloride sensor to the chloride ions depends on the concentration of interfering species, such as hydroxide and sulphide ions in the medium. These processes subsequently limit the rate of reactions at the sensor’s surface. A better insight into these parameters is important for understanding the chloride sensor’s response (Figure 1). Therefore, the cement chemistry and composition of cementitious materials are reviewed in the following sections.

In view of the factors outlined above, this paper follows a sequence of steps to determine chloride ions’ and sensors’ responses in a concrete environment. Figure 1 can be considered as a schematic presentation of the different influencing parameters, interfaces and points of interest with regard to a Ag/AgCl sensor performance. This work encompasses five main sections, structured as follows:

- In the first section, the main hydration products of cement paste in the bulk matrix and at the sensor–cement paste interface are described. The composition of the pore solution of cementitious materials, i.e., the various ions in contact with the sensor’s surface and hence of importance for sensors’ readings, is also assessed.
- Next, the classification of chloride ions in cementitious materials is reviewed and the importance of chloride sensors for local chloride measurements, specifically at the level of the reinforcement, is discussed.
- Section 4 presents the different techniques for the determination of chloride content in concrete and the advantages of chloride sensors in particular for free chloride determination.
- The next section bridges fundamental considerations and technical background, both related to the performance, characterization and application of Ag/AgCl sensors. The advantages and drawbacks of the chloride sensors are discussed. The thermodynamic behaviour of silver and the electrochemical kinetics of AgCl formation are discussed. Then, the relevance of these parameters to the reliability of the sensors’ response in cementitious materials is evaluated. The above framework outlines the structure of this paper, highlighting the principles, related mechanisms and determining factors for monitoring of chloride ions in cementitious materials.

## 2. Important Properties of Cementitious Materials in View of Chloride Measurements via Ag/AgCl Sensors

A cementitious paste is a heterogeneous material with high alkalinity, a pore solution with different composition, and a pore system with different porosity and pore size distribution (Figure 1d,e) [3]. In the following, some properties of cementitious materials that are important for the response of chloride sensor are presented. The main hydration products and microstructure of cementitious materials are described (Section 2.1). Moreover, the concentration of interfering ions, i.e., hydroxide and sulphide ions (Figure 1b) in the pore solution of Portland cement paste and blast furnace slag cement paste is presented (Section 2.2). The slag cement paste is considered because it contains a high amount of water soluble sulphates, and sulphide ions [4].

### 2.1. Hydration Products and Microstructure of Cement Paste

Hardened Portland cement paste is a porous material containing calcium hydroxide (Ca(OH)_2_, Portlandite), aluminate compounds, such as monosulphate, hydroxyl-AFm, ettringite (AFt) and unhydrated cement particles, surrounded by an amorphous hydration product, known as C-S-H (calcium silicate hydrate) [5].

The heterogeneity of hydration products and the microstructure of cementitious materials are important factors in view of the reliability and reproducibility of the sensor’s response (Figure 1d,e). If these factors are not considered, the performance of a chloride sensor will remain uncertain. These concerns are currently not well understood. However, they can be assessed from the available studies on the interfacial properties between the cement paste and the aggregate. The microstructure and hydration products of cement paste in the vicinity of an aggregate differ from those further away from the aggregate. This distinctive region is termed the interfacial transition zone (ITZ) [5]. When the cement grains encounter the “wall” of the aggregate, a region of higher porosity and different composition near the aggregate surface appears [6]. The porosity of ITZ is two to three times higher than the porosity in the bulk [7]. The wall effect creates a water concentration gradient around the aggregate, i.e., a w/c ratio gradient. A locally higher w/c ratio with fewer nucleation sites forms larger and preferentially oriented crystals in contact with aggregates [7]. In this region, ettringite and calcium hydroxide tend to form larger crystals in oriented layers [5]. A rim of massive calcium hydroxide can often be observed around the aggregates. This implies that the ITZ properties at the interface between the sensor and cementitious materials are the principal factor influencing the performance of the chloride sensor.

### 2.2. Ions in Pore Solution

As shown in Figure 1b, the composition of the pore solution of cementitious materials represents the ions that are in contact with the sensor. Hydroxide and sulphide ions are the two main ions of cement paste that can interfere with the chloride sensor’s response. In the following, the concentration of these ions in the pore solution of Portland cement and slag cement is reviewed.

#### 2.2.1. Portland Cement

The aqueous electrolyte in the spaces not filled by solid hydration products is known as the pore solution [8]. The chloride sensor in cementitious material, similar to embedded reinforcement, is in contact with the pore solution. The composition of the pore solution depends on the hydration of different cement phases and solubility of these products. Once cement is mixed with water and starts to dissolve, every element that is present in the cement can also be found in the form of dissolved ions in the pore solution.

The pore solution of Portland cement paste is mainly composed of alkaline hydroxides (mainly KOH and NaOH). Other species, such as SO_4_^2−^ and Ca^2+^, are present in much lower concentrations [9,10,11,12].

The change in the alkalinity of the pore solution during cement hydration depends on the w/c ratio and cement type [13]. Alkalis in Portland cement (K_2_O and Na_2_O) play a significant role for the high pH range of the pore solution (13.2 to 13.8) [14]. The hydroxide concentration in cement paste with CEM I 42.5N and w/c = 0.5 increases from 150 mM at 7 h to 540 mM after 29 days [13].

#### 2.2.2. Blast Furnace Slag Cement

The alkalinity of the pore solution in slag cement paste (CEM III/B, w/c = 0.5, pH = 13.1) is lower than that of Portland cement paste (CEM I, w/c = 0.5, pH = 13.9) [3]. For slag cement, the concentration of alkaline ions (Na^+^ and K^+^) and hydroxide ions decreases during the slag hydration process. This results in a pH reduction of the pore solution for slag cement paste, to a value lower than that of Portland cement paste [3,15].

The pore solution composition of slag cement paste is to a large extent similar to that of Portland cement paste. The difference is a remarkable amount of sulphide ions (S^2−^) in slag cement paste [4]. The sulphide content in slag is about 1%, while in Portland cement it is lower than 0.1% [16]. After 10 h of hydration, sulphide ions were detected in the pore solution of blended cement paste containing slag (CEM III/B 42.5 L) [4]. The concentration of sulphide ions increased to 5 mM after seven days, and then stabilized at 8–11 mM [4]. The concentration of hydroxide and sulphide ions is important for the performance of an Ag/AgCl chloride sensor (Figure 1b), as will be reviewed and discussed in Section 6.

## 3. Chloride Ions in Concrete

### 3.1. Classification of Chloride Ions

**Figure 2:**
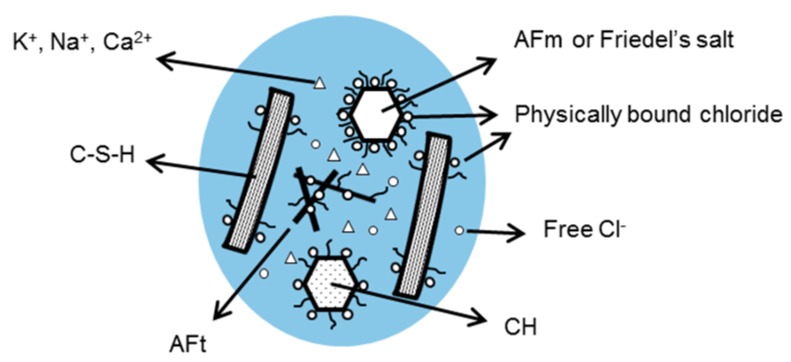
Schematic of main hydration products of cement paste and their contribution to the binding of chloride ions.

The ingress of chloride ions in concrete takes place via the pore system and can be affected by the binding of chloride ions to the hydration products. The well-known classification of chloride ions in cement-based materials is that of chemically bound chloride, physically bound chloride and free chloride ions. The chloride ions can be bound chemically in compounds like Friedel’s salt (calcium chloroaluminate hydrate) or adsorbed physically at the surface of cement hydration products, such as C-S-H, Friedel’s salt and Portlandite. The chloride ions in the bulk pore solution are called free chloride (Figure 2). The binding of chloride ions to the cement hydration products continues up to a level at which an equilibrium between bound (chemically and physically) and free chlorides is reached.

Glass et al. [17] classified the chloride ions in the concrete pore solution into four categories—free, loosely bound, bound and strongly bound chlorides. The amount of free chloride can be measured by the “equilibrium method”. In this method, concrete samples are stored in a solution of known chloride concentration until equilibrium is reached between the external solution and the concrete pore solution. For example, the time required for a cement paste sample with 1 cm thickness to reach equilibrium can extend up to a year. In equilibrium condition, the free chloride in the concrete sample is equal to the chloride concentration in the external solution. The chloride is “loosely bound” when it can be extracted from the concrete samples under high pressure (in the range of hundreds of MPa). The physically bound chloride can be considered as loosely bound chloride. The term “bound chloride” refers to the chloride that cannot be extracted from the concrete samples under pressure. This holds for the chemically bound chloride. The traditional method for determination of total chloride content in a concrete sample is “acid-soluble chloride extraction” [18,19,20]. Extraction of total chloride content using this method may not end with release of all the bound chlorides. Therefore, the total chloride content of the sample can be underestimated [21]. The part of bound chloride that is not released by the acid-soluble extraction method is known as “strongly bound chloride”. Although most of the phases containing chloride compounds dissolve in the acidic medium of the test [18,19,20], the release of “strongly bound” chloride may require a higher acidity.

### 3.2. Free Chloride

There are several techniques for the evaluation of the free chloride content. Different test procedures, applied in these techniques, result in different outcomes for the free chloride content. For instance, chemical extraction of chloride ions from a concrete sample gives a significantly higher amount of chloride than extraction under high pressure [22]. Determination of free chloride content is discussed in Section 4.2 with respect to chloride sensors’ response.

Section 3.3 reviews main aspects of the classification of bound chlorides in a cementitious matrix. These are important in view of the general debates about the determination of chloride content and also the sensors’ application. The available techniques for the determination of chloride content in cementitious materials are described in Section 4.

### 3.3. Bound Chloride

#### 3.3.1. Chemical Binding of Chloride

Friedel’s salt is the main reaction product of chemical binding of chloride ions in concrete. It forms due to the reaction between the chloride ions and hydration products of C_3_A, e.g., hydroxyl-AFm, monosulphate and AFt phases [23]. It is assumed that all aluminate hydrates transform to Friedel’s salt with increasing chloride concentration in the pore solution [24]. In general, the tendency of sulphate ions (SO_4_^2−^) to bind in hydration products is higher than that of chloride and hydroxide ions, i.e., SO_4_^2−^ > Cl^−^ >> OH^−^ [25]. However, the concentration of sulphate ions (SO_4_^2−^) in the pore solution of mature (28 days) cement paste is low (Table 1). Therefore, chloride ions can react with the AFm phases to form Friedel’s salt. Even the sulphate-containing hydration products (monosulfate and Aft) convert to Friedel’s salt if the chloride concentration in the pore solution is sufficiently high [26].

#### 3.3.2. Physical Binding of Chloride

Similar to most other materials, the surface of hydration products becomes electrically charged when in contact with a polar solution such as water. The ionic binding properties of hydration products govern the dissolution of surface species or adsorption of ions from the solution. This process electrically charges the surface of the hydrated phases.

**Figure 3:**
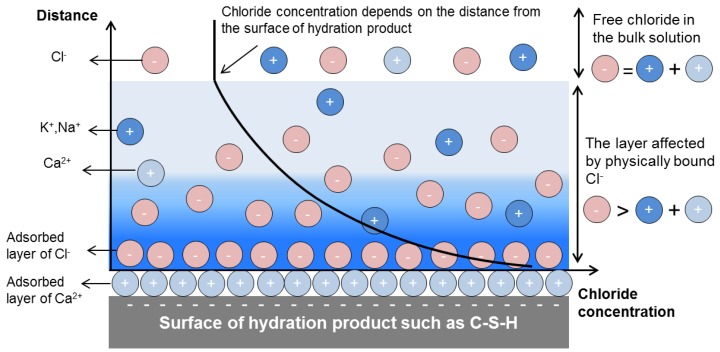
Schematic diagram of physical binding of chloride ions to the cement hydration product exposed to chloride environment. The adsorption of chloride ions subsequently induces a chloride concentration gradient between the bulk solution and the surface of a hydration product.

If we look at the interface of a hydration product, we see an accumulation of chloride ions and a depletion of cations near the charged surface of hydration products and in the vicinity of adsorbed layer of cations (Figure 3) [27,28]. In the bulk solution, electroneutrality prevails (Figure 3) [28]. Although the layer of physically bound chloride into the solution is not more than a few nm, it can comprise a considerable amount of chloride ions [9].

As mentioned in Section 3.1, the physically bound chloride (Figure 3) can be “squeezed out” when the cement paste is subjected to high pressure and/or thermal loads [17,29]. Therefore, the physically bound chloride has been classified as a loosely bound chloride [17]. The erratic random movement of physically bound chloride is continuously affected by the surrounding molecules [30]. In this condition, the activity of chloride ions increases with increasing distance from the surface of hydration product (Figure 3) [30,31]. Therefore, physically bound chloride can be considered as free chloride ions, but with low activity.

The chloride binding mechanisms and microstructure of cementitious materials are also important for determination of the so-called chloride threshold value. The threshold value, i.e., the value of the critical chloride content for the corrosion initiation of reinforcing steel, is still heavily debated. In the next section, several aspects of determination of the chloride threshold value are presented.

### 3.4. Chloride Threshold Value

The process of steel corrosion in reinforced concrete can be divided into two phases, initiation and propagation [23,32,33,34]. The first stage, i.e., initiation, is generally related to the time needed for a critical chloride concentration to reach the steel bar and initiate corrosion. The subsequent propagation stage extends up to the time when the corrosion damage is beyond the acceptable limits and cracks due to the expansion of corrosion products appearing on the concrete surface.

An impressive number of studies on the chloride threshold value have been published since the 1960s [35]. As shown in Table 2, a unique chloride threshold value does not exist. Similarly, a generally accepted or standardized method for determination of the critical chloride content does not exist as well. Even the concept of chloride threshold value has been questioned [36,37]. This controversy is mainly due to the variety of influencing factors, such as concrete mixture, environmental factors and surface condition of the embedded reinforcing steel. Apart from these factors, different techniques and criteria have been used for both the detection of the time to steel depassivation and measuring the chloride content close to the reinforcement. The measured chloride content is presented in different units and related to the concrete or binder weight (Table 2). As the binder content is not always known, it is sometimes preferred to present the total chloride content as a percentage of the weight of concrete. The free chloride content is presented in different ways: as a percentage of the binder or concrete weight, as mole per litre of concrete pore solution (mol/L), or as the ratio of chloride to hydroxide ions ([Cl^−^]/[OH^−^]) in the pore solution (Table 2).

Some researchers claimed that the chloride threshold value should be expressed as the total chloride content [34]. Many authors believe, however, that bound chloride does not impose any risk for corrosion initiation [50]. Still, the chloride threshold value is quantified in terms of either total or free chloride content (Table 2).

Actually, measuring the free chloride content in the cement paste, mortar or concrete is complicated. This is mainly due to the limitations of the available techniques for extraction of free chloride ions from the cementitious materials. As a result, the total chloride content is generally used for representation of the chloride threshold value.

**Figure 4:**
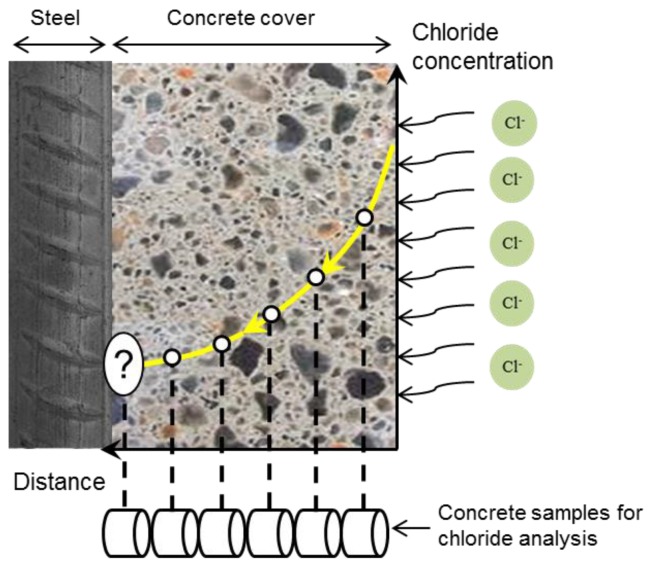
Chloride profile in concrete and determination of chloride threshold value.

The different methods for the determination of chloride content also affect the variation in the reported chloride threshold values. Traditionally, one or more cores from the concrete cover are taken at the time of corrosion initiation (Figure 4). The sliced cores are analysed for chloride at different depths from the concrete surface. The chloride content in the slice near the rebar depth is compared with the chloride threshold value. The measured chloride content in this relatively large sample is not exactly representative of the local amount of chloride close to reinforcement. The chloride content in the sample along the rebar–concrete interface is the average amount of chloride over the rebar surface. To determine the deviation from the average value, it is reasonable to measure the chloride content in local points at the steel-concrete interface [51]. In this regard, non-destructive in situ techniques have been developed for the determination of the local chloride content at different places close to the reinforcement. In the next section, available techniques and main limitations for measuring the local chloride content are discussed and the significance of developing a chloride sensor for chloride measurement is explained.

## 4. Techniques for Determination of the Chloride Content in Concrete

### 4.1. Lab Techniques

**Figure 5:**
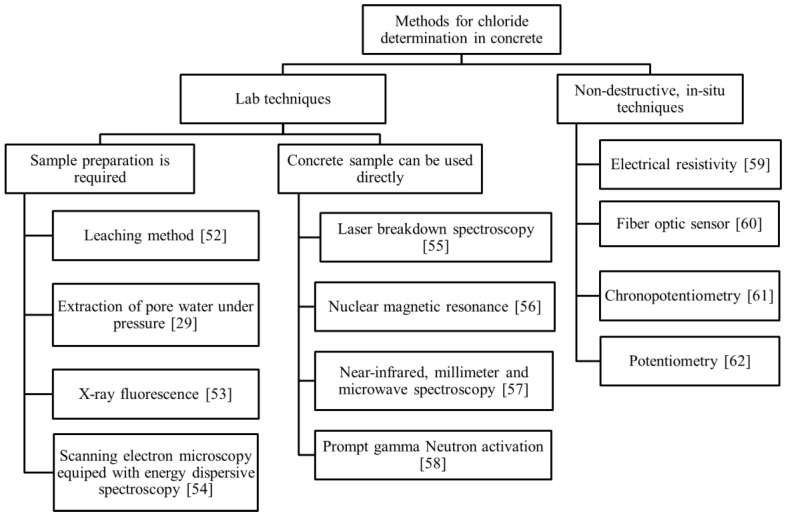
An overview of the available techniques for measuring the chloride content in concrete [29,52,53,54,55,56,57,58,59,60,61,62].

The techniques for determination of the chloride content in concrete can be classified into lab techniques and non-destructive in situ techniques. Figure 5 gives an overview of these techniques. In lab techniques, the concrete samples/cores are taken from the structure for further analysis in the laboratory. Generally, the lab techniques are time-consuming, expensive and cannot be used for continuous monitoring of the chloride content in a concrete structure.

The most popular and traditional lab technique for chloride determination in cementitious materials is the leaching method. In this method, acid-soluble chloride and water-soluble chloride are extracted from the powdered concrete samples into the solution. The extracted solution is further analysed for chloride content using different methods, such as Volhard titration [19], potentiometric titration [63] and photometric analysis [64]. In most cases, the acid-soluble chloride is equivalent to the total chloride [18]. Water-soluble chloride does not necessarily represent the free chloride only, as it is sensitive to the test condition. The extracted chloride content depends on the fineness of sample powder, the amount of water added to the powder (powder/water ratio), the contact time and the temperature of the suspension [17,51,65]. In fact, it was claimed that, given enough time and water, all the chlorides can be extracted from the sample [66]. As a result, the different values of water-soluble chloride in a concrete sample can partly be attributed to the different test procedure used [67,68].

The traditional method for the determination of free chloride content in concrete specimens in the lab is extraction of pore water under pressure. The concrete specimens are pressed to extract the pore water. A few millilitres of pore water are needed for chloride analysis [29]. To obtain the minimal volume of pore water, a large volume of concrete sample with high w/c ratio, low content of aggregate and wet moisture state was suggested [69]. This technique is not practical for field application. Moreover, local chloride gradients cannot be determined with this technique.

The total chloride content can also be measured by X-ray fluorescence, scanning electron microscopy equipped with energy dispersive spectroscopy and laser breakdown spectroscopy. The first two techniques need preparation of concrete sample/core before chloride measurement. The advantage of chloride determination using laser breakdown spectroscopy is the possibility for on-site application and no need for sample preparation.

Other lab techniques are nuclear magnetic resonance (NMR), prompt gamma neutron activation analysis and near-infrared, millimetre and micro-wave spectroscopy. These techniques are expensive and labour-intensive, so their use is limited to appropriately equipped laboratories.

### 4.2. Non-Destructive In Situ Techniques

In the past decades attempts were made to develop non-destructive techniques for in situ monitoring of the free chloride ions and chloride profiles in concrete structures. Concrete is a heterogeneous material with high alkaline pore solution, different composition and pore systems with various porosity and pore size distribution [3]. The many influencing factors make the application of in situ techniques complex and difficult.

In situ techniques for measuring, or indicating, the chloride concentration are: electrical resistivity, fibre optic sensors, chronopotentiometry and potentiometry methods. The electrical resistivity can be used not only in field structures, but also in the laboratory on samples taken from the structure [65]. However, resistivity measurements for cement-based material are very sensitive to moisture content, while not as sensitive to the chloride content. Therefore, alterations in the concrete resistivity measurements cannot simply be correlated to the chloride content in the pore water.

A fibre optic sensor consists of a fibre with an optical transducer, sensitive to chloride ions. The lifetime of optical transducers, protection of fibres and the bulky measurement setup are the limitations of this technique [60].

Chronopotentiometry is a dynamic electrochemical method used for an indicative determination of the chloride content at the surface of a working electrode. The current stimulus is applied to the working electrode and the potential response is measured against a pseudo-reference electrode. The use of this method for chloride measurement in concrete was recently hypothesized [61].

**Figure 6:**
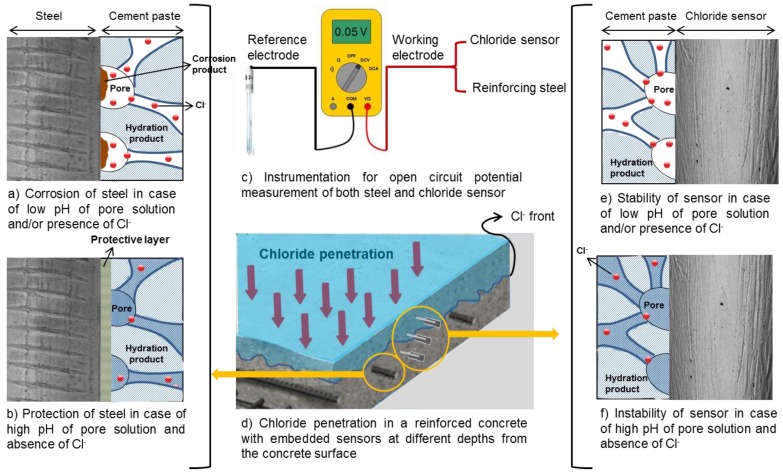
Schematic representation of chloride sensor and steel rods in cementitious materials. The OCP of chloride sensor can be related to the chloride content in the medium, while the OCP of steel rod represents the electrochemical state of the steel in concrete.

The potentiometry technique is a measurement of open circuit potential (OCP) of an embedded Ag/AgCl electrode (chloride sensor) against a reference electrode (Figure 6c). This method is considered the most practical approach for continuous monitoring of chloride content in the concrete environment. Interpretation of the measured OCP requires detailed information of the environment at the sensor–concrete interface (Figure 1d,e and Figure 6e,f) and knowledge of characteristics of the chloride sensor (Figure 1a,b). In this regard, the composition of the pore solution is important for the potential response of both the chloride sensor and reinforcing steel rod (Figure 6). For example, the concentration of hydroxide ions can affect the stability of the chloride sensor. The chloride sensor is generally not stable in an alkaline medium with low chloride concentration [62,70,71] (Figure 6f). The stability of chloride sensor increases when chloride ions are present in the pore solution (Figure 6e) [62]. In addition, in low alkaline mediums, the interference of hydroxide ions with the sensor response is insignificant [72,73]. A cement-rich layer at the steel–concrete interface contributes to the formation of a protective layer on the surface of the reinforcement (Figure 6b) [74]. Under certain circumstances the protective layer can be disrupted, causing active corrosion. The reinforcing steel corrodes uniformly if the alkalinity of the medium decreases to values below pH 9 [23]. However, the hydroxide concentration is not the only factor determining steel activity. As mentioned, chloride ions are well-known corrodents and cause steel de-passivation upon reaching a certain concentration (threshold). It was suggested that the ratio [Cl^−^]/[OH^−^] is a better criterion to evaluate the chloride content for corrosion initiation [35,38]. Although the pH of the pore solution is not the only parameter affecting the chloride threshold value (as discussed in Section 3.4), and although chloride ions, in fact, do not alter the pH of the pore solution, pH affects the chloride ions’ activity towards corrosion initiation [38,75,76]. It was reported that the effect of [Cl^−^]/[OH^−^] ratio on the corrosion initiation should be considered with respect to the other influential factors, such as the physical condition of the steel/concrete interface [67,68]. Therefore, a higher [Cl^−^]/[OH^−^] ratio at the level of reinforcement does not necessarily represent a higher risk of corrosion initiation [77].

In the previous sections, the main properties of cementitious materials for the sensor’s measurement were discussed. The obtained information revealed the importance of microstructure of cementitious materials at the interface with the sensor for a reliable sensor reading (Figure 1d,e). In the next section, the importance of the chloride sensor’s characteristics in the presence of interfering ions is discussed (Figure 1a,b).

## 5. Working Principles of the Ag/AgCl Electrode

Although the instrumentation for the Ag/AgCl chloride sensor seems to be simple (Figure 1c and Figure 6c), the interpretation of the measurements requires knowledge of the electrochemical state of the chloride sensor itself and the interaction of the sensor with the environment.

Similar to all electrochemical phenomena, the response of an Ag/AgCl chloride sensor follows the laws of thermodynamics. Therefore, an overview of the thermodynamically plausible reactions and different oxidation states of Ag in highly alkaline solutions will be presented. A clear understanding of the sensor’s response requires knowledge of the kinetics of the reactions at the sensor’s surface. As a result, kinetic parameters and relevant constraints will be also discussed in the next section.

Silver in the form of an Ag/AgCl electrode is widely used in industrial technologies, medical instruments and as a reference electrode in electrochemistry. Some state-of-the-art reports on Ag/AgCl electrodes date back to 1900 [78]. However, the relation between the AgCl structure, the resistivity of the AgCl layer and the transport process of silver and chloride ions in the layer has not been sufficiently described [79]. Consequently, debates are still going on as to how to interpret the sensor readings.

**Figure 7:**
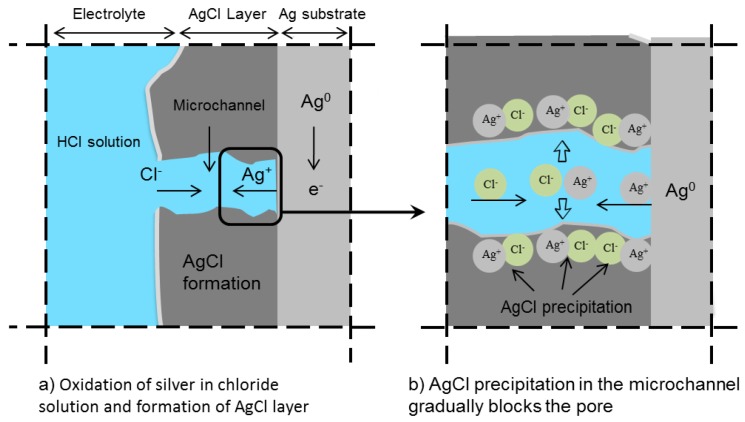
The electrochemical oxidation of Ag in HCl solution.

One of the characteristics of an Ag/AgCl electrode is its (supposedly) rapid response. When no kinetic restrictions apply, the dynamic equilibrium between metallic silver (Ag^0^) and Ag^+^ can be established in a short period. This feature makes the noble Ag metal prone to “corrosion” in environments with aggressive ions, such as chloride ions. The reaction rate depends on the electrochemical state of the electrode in the aqueous medium. The electrochemical oxidation of silver in chloride-containing solutions, e.g., HCl solution, results in the formation of a silver chloride layer on the silver substrate (Figure 7) [79]. The mechanism of silver chloride formation (Figure 7) is discussed in Section 6.1.

The electrochemical response of the Ag/AgCl electrode in a certain environment is different from the Ag metal alone. The change in activity of chloride ions (concentration) in the medium subsequently affects the equilibrium at the Ag/AgCl interface. However, the electrode returns rapidly to the equilibrium potential after a small transient perturbation. The principle of Ag/AgCl electrode response is based on two equilibriums: the electrochemical equilibrium involving the formation of interfacial potential, and the solubility equilibrium between the Ag cation and its sparsely soluble salt (AgCl) [80]. This is further explained in the following.

The electrochemical equilibrium at the silver surface can be described by Equation (1):

Ag ↔ Ag^+^ + e^−^. (1)

At 25 °C, the solubility product (K_sp_) of AgCl is K_sp_ = 1.8 × 10^−10^ [81].

The solubility equilibrium is in accordance to Equation (2):

AgCl ↔ Ag^+^ + Cl^−^; (2)

combining the above two reactions, the oxidation–reduction equilibrium reaction can be written as:

AgCl + e^−^ ↔ Ag + Cl^−^, (3)

in which solid AgCl is deposited at a potential near the thermodynamic reversible potential for the Ag/AgCl electrode in the chloride-containing medium. In this description, the silver chloride is the “core” of Ag/AgCl electrode (chloride sensor), controlling its selectivity for the chloride ions. The relation between the half-cell potential of the chloride sensor and the chloride activity is expressed by the Nernst equation [62]:

(4) $$E_{\mathrm{Ag}/\mathrm{AgCl}}=E_{\mathrm{Ag}/\mathrm{AgCl}}^{0}-2.303\frac{\mathrm{RT}}{\mathrm{nF}}\mathrm{lg}[a_{{\mathrm{Cl}}^{-}}],$$

where E_Ag/AgCl_ is the measured electrode potential [V], E°_Ag/AgCl_ is the standard electrode potential of the Ag/AgCl electrode [V], *a*_cl_^−^ is the activity of the chloride ions [mol∙dm^−3^] in the vicinity of the electrode, R is the gas constant [J∙mol^−1^∙K^−1^], F is the Faraday constant [C∙mol^−1^] and T is the absolute temperature (K).

The chloride activity is linked to the chloride ions concentration by the activity coefficient (γ) [82,83,84]:

(5) $$a_{{\mathrm{Cl}}^{-}}=C_{{\mathrm{Cl}}^{-}}\cdot \gamma_{{\mathrm{Cl}}^{-}}$$

Therefore, by measuring the potential of the Ag/AgCl electrode and using the Nernst equation, the chloride activity, and subsequently the chloride concentration in the solution can be calculated.

## 6. Discussion

### 6.1. Electrochemical Kinetics of AgCl Layer Formation

In most studies, anodization of silver in chloride-containing solutions was the method used for the oxidation of silver and formation of AgCl on the silver substrate [85,86]. The main differences among the anodization regimes are the current density and the duration of anodization. In some cases, the anodized AgCl layer was additionally dipped in an AgCl melt to achieve a more stable AgCl layer.

**Figure 8:**
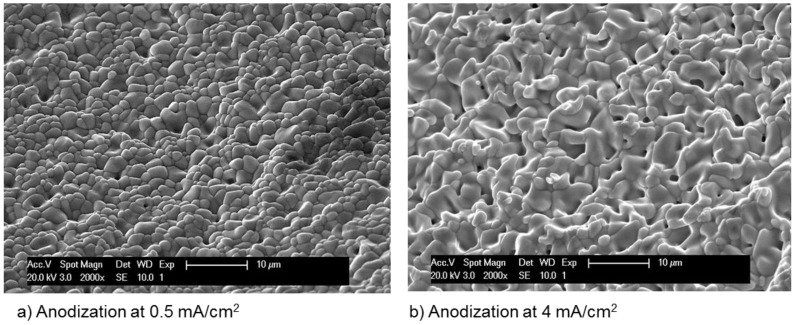
Surface morphology of an AgCl layer prepared by one-hour anodization at different current densities.

It has been reported that after nucleation, the growth of an AgCl layer on the silver substrate proceeds with the formation of small patches of rounded and smooth surface with no sign of crystal orientation [87]. At this stage, the size of the AgCl particles is less than 0.5 μm [88]. However, transition to a multilayer brings dense and fine AgCl particles to the surface (Figure 8a). Anodization at high current density changes the morphology of the AgCl top layer to a so-called mosaic appearance (Figure 8b).

The galvanostatic growth of a AgCl multilayer is accompanied by an increase in the overpotential due to the ohmic resistance of the AgCl layer itself [89,90]. The AgCl itself is non-conductive, so the effective conductivity of such a layer depends on the pores (microchannels) between the AgCl grains (Figure 7a). Therefore, the growth of the AgCl layer is a function of the ionic conductivity of the microchannels [91]. Both low and high field conduction mechanisms are reported for the growth of an AgCl layer [79]. When the AgCl layer is porous and the overpotential is low, the ionic conductivity takes place through microchannels (extrinsic conductivity). This is known as low field conduction.

At high overpotential, when the relation between the current density and the potential is exponential, microchannels may become blocked. As a result, ions migrate through the solid AgCl particles (intrinsic conductivity). This is known as high field conduction [92]. The importance of the above mechanisms for the application of the Ag/AgCl electrode as a chloride sensor is related to their subsequent influence on the AgCl layer morphology and microstructure.

The observed potential difference among the Ag/AgCl chloride sensors in an alkaline medium with the same chloride concentration was attributed to the sensor preparation method [93,94]. However, no evidence in support of this assertion was provided. For instance, the OCPs of two differently prepared Ag/AgCl chloride sensors were measured in Ca(OH)_2_ solution with 0.002 M to 0.86 M NaCl. The observed OCP of the sensors were different in the entire range of the chloride concentrations. The effect was higher at lower chloride concentration, e.g., 60 mV at 0.002 M chloride concentration versus 40 mV at 0.86 M chloride concentration [95]. In a similar study, two differently prepared chloride sensors were embedded in mortar samples. A difference of 100 mV in the initial OCP of the chloride sensors was reported [93]. These studies did not provide further evidence for the observed discrepancies between the sensor readings. Thus, the influence of AgCl layer properties on the reproducibility and reliability of the sensor’s measurements was not further discussed [93,94].

Within anodization, the mechanism of ionic conductivity governs the kinetics for silver dissolution and AgCl formation. The exchange in this mechanism from extrinsic to intrinsic (see paragraphs 3 and 4 of this section) can subsequently affect the AgCl layer morphology and microstructure. The alteration in the physical properties of the AgCl layer (porosity, thickness, morphology, etc.) can partly affect the stability and reliability of the sensor’s measurements in cementitious materials.

### 6.2. Thermodynamic Behaviour of Silver

AgCl is a “corrosion product” of silver in a chloride-containing environment. The electrochemical state of silver, similar to all electrochemical phenomena, follows laws of thermodynamics. A full understanding and prediction of the corrosion process undoubtedly requires a consideration of thermodynamic principles. Following these principles, the standard Gibbs free energy (∆G) for a given compound is the energy for the formation of 1 mole of that compound from its constituents. A negative ∆G is an indication of a spontaneous reaction.

Table 3 gives the standard Gibbs energies of formation of the species and phases in the silver–chloride–water system [96]. The positive Gibbs energy of silver ions (Ag^+^) and AgO is an indication of non-spontaneous reactions. The Gibbs energy of the ionic silver compounds, such as Ag(OH)_2_^−^, AgCl_2_^−^ and AgCl_4_^3−^ is more negative than the crystalline phases (c), such as AgCl (c) and Ag_2_O (c). Consequently, the formation of the former compounds is more product-favoured than the latter ones. The spontaneous corrosion product of silver in a chloride-containing medium is AgCl (e.g., AgCl with Gibbs energy = −110 KJ/mol), rather than silver oxide (e.g., Ag_2_O with Gibbs energy = −11 KJ/mol).

Kinetic restrictions exist in all thermodynamically possible reactions. The actual occurrence, direction and rate of thermodynamically possible reactions are governed by electrochemical kinetics. For example, the thermodynamic data indicates the formation of ionic silver compounds or AgCl as the favoured reaction product on silver. However, the rate of formation of these compounds depends on the concentration of chloride and hydroxide ions in the medium. This process is also reflected in the response of Ag/AgCl sensor in an alkaline medium with low chloride content. In this condition, the alkalinity of the medium and transformation rate of silver chloride to silver oxide at the sensor’s surface governs the sensor’s response. The influence of this transformation rate on the sensor’s response is generally not discussed in the reported studies.

The Pourbaix (potential—pH) diagram is based on thermodynamics, so it can only “map out” thermodynamic stability of metal species and compounds at various combinations of equilibrium potential and pH in an aqueous medium under standard conditions. Brief thermodynamic principles for the behaviour of silver in water in the presence and absence of chloride ions are discussed in what follows.

The Pourbaix diagram for the silver–chloride–water system [97] shows that the chloride ion is a strong oxidizing agent for Ag(s) and crystalline AgCl (AgCl (c)) is the thermodynamically favourable oxidation product. It is reported that the increase in the concentration of chloride ions causes dissolution of AgCl (c) and formation of ionic silver chloride, such as AgCl_2_^−^ or AgCl_4_^3−^ [98]. In chloride-containing alkaline medium, the silver oxide compounds can thermodynamically exist. Silver chloride can counteract silver oxide formation, depending on the chloride concentration and pH of the environment. All these processes can be related to the performance of the Ag/AgCl chloride sensor in cementitious materials. The dissolution of AgCl (c) and formation of silver chloride complexes (AgCl_n_^(1−n)^) or formation of silver oxide may deviate the response of the chloride sensor from the Nernstian behaviour. Therefore, the potential measured as E_Ag/AgCl_ will no longer satisfy Equation (4) or be valid as such.

### 6.3. Considerations with Regard to Chloride Ions’ Activity and pH

It is known that the presence of AgCl(c) is essential for the Nernstian behaviour of the sensor. The stability of silver chloride is a function of silver and chloride activity at the sensor’s surface as well as the pH of the medium. For example, the stability of AgCl(c) is limited to a certain range of chloride activity (10^−6^ < ɑ_cl_ <1) [99]. Trespassing the limit, products other than silver chloride form, which undeniably affect the performance of the sensor. The silver oxide complex ions, or silver chloride complexes, are not necessarily attracted to the sensor’s surface [100]. The ions can leave the sensor’s surface and diffuse into the environment (e.g., within the cementitious materials). In this regard, the properties of cementitious materials, such as porosity, are important. This process can subsequently shift the sensor’s OCP towards values that cannot be expected from the Nernst equation for an Ag/AgCl electrode. Therefore, the AgCl layer properties and the Ag/AgCl interface may influence the stability and reliability of the sensor’s response. This should be taken into account when long-lasting performance and ease of application of chloride sensors in cementitious materials are desired.

### 6.4. Reliability of Sensor Response in Cementitious Materials

**Figure 9:**
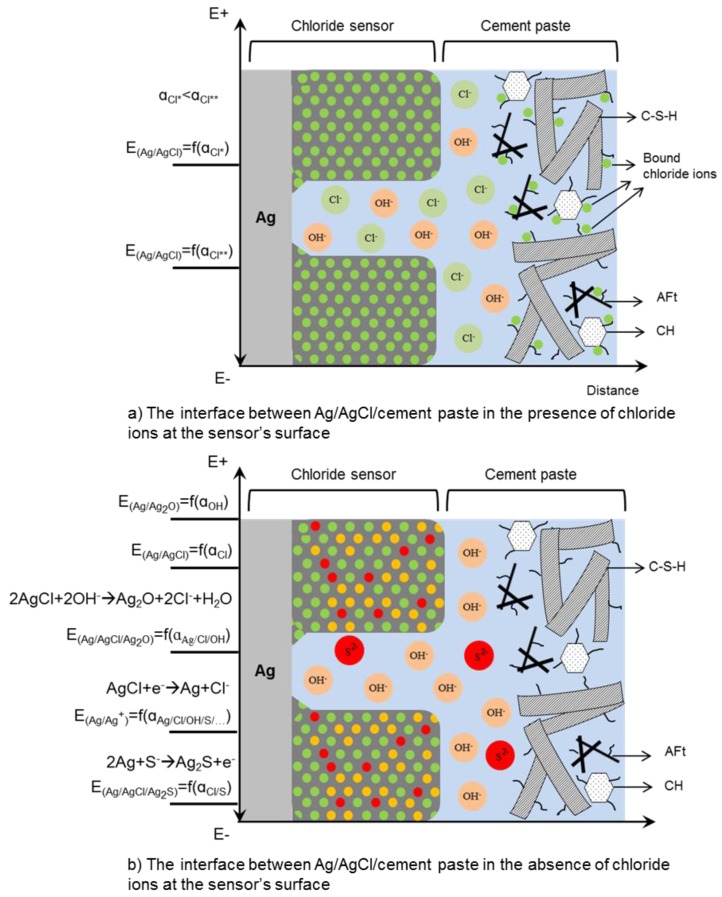
Schematic of Ag/AgCl/pore solution interface in cementitious materials. (**a**) In the presence of chloride ions, the surface of the sensor mainly consists of AgCl particles. The binding of chloride ions to the hydration products together with the microstructure of cementitious materials affect the activity of the chloride ions at the sensor’s surface; (**b**) In the absence of chloride ions, gradual dissolution of AgCl layer is accompanied by formation of Ag_2_O, Ag^0^ or Ag_2_S (in the case of slag cement concrete) [101,102,103]. The formation of these products depends on the concentration of hydroxide and sulphide ions in the medium and the microstructure of cementitious materials at the interfacial zone with the sensor.

The performance of a chloride sensor in cementitious materials depends on the thermodynamic stability of the AgCl layer at the sensor’s surface. In the presence of chloride ions in the solution, AgCl is the dominant reaction product (Figure 9a). However, the interference of hydroxide and sulphide ions in the solution can limit the otherwise plausible reaction of AgCl formation on the sensor’s surface (Figure 9b). Therefore, the rate of AgCl transformation into Ag_2_O or Ag_2_S controls the variation in the sensor’s OCP. This process essentially makes the potential of the Ag/AgCl sensor deviate from the expected Nernstian response (as would be expected from Equation (4)).

In a solution with high pH and low chloride concentration (Figure 9b), AgCl can be partly or completely converted into silver oxide compounds (Ag_2_O, AgO, AgOH, etc.). Therefore, the sensor gradually acts as a pH sensor rather than a chloride sensor. In this condition, the activity of silver ions in the vicinity of the sensor is determined by exchange equilibrium and formation of Ag_2_O, as shown in Figure 9b. However, the dissolved silver chloride and/or the formed silver oxide ions (K_sp_ (AgOH) = 2 × 10^−8^ at 25 °C [104]) do not necessarily adhere to the sensor’s surface [100]. These ions can leave the sensor’s surface and diffuse into the cementitious material.

Thermodynamically, the formation of Ag_2_S with lower solubility (K_sp_ (Ag_2_S) = 1.6 × 10^−49^ at 25 °C) is favoured when a sufficient amount of sulphide ions is in the medium, e.g., in concrete with slag cement. It has been stated that the sulphide content of higher than 6 mM can affect the chloride sensor’s response in a model solution [103]. Such a small content of sulphide ions can significantly change the sensor’s response. The extent to which Ag_2_S contributes to the product layer at the sensor’s surface determines the cathodic potential shift of the sensor (Figure 9b) [105]. The interference of sulphide ions with the chloride sensor is more serious than that of hydroxide ions [101,106]. This is mainly because of very small solubility product of silver sulphide (as above mentioned). It is strongly suggested that the sulphide ions in the pore solution of slag cement may affect the chloride sensor’s measurements [103]. This assertion cannot be generalized for the application of the chloride sensor in concrete with medium/low slag cement (e.g., CEM III/A). In such a medium, the kinetics of reactions of sulphide ions with the AgCl layer at the sensor’s surface should be considered.

The AgCl layer gradually dissolves in chloride-free solutions, even in the absence of interfering ions (hydroxide and sulphide ions). The gradual dissolution of the AgCl layer can be fatal for the sensor performance [107]. The dissolved ions can gradually diffuse into the medium, exposing the Ag substrate to direct contact with the environment. In this condition, the exact behaviour of the Ag is difficult or even impossible to predict, when the sensor is under “mixed potential control”. The OCP of the Ag metal is unstable, irreproducible and not theoretically meaningful. The measurements depend on adventitious impurities in the medium (Figure 9b) [108]. Therefore, the potential response of silver metal in cementitious materials cannot represent the alteration in the concentration of a certain ion, i.e., chloride in the medium.

All the above considerations with regards to (electro) chemical reactions and actual sensors’ response depend on the availability of ions (chloride ions and interfering ones) and ion transport, or limitations in the surrounding medium, i.e., pore water and pore network.

The importance of the microstructure of cementitious materials involves the influence of different hydration products and their compactness at the sensor’s surface. In the presence of large pores at the sensor–cement paste interface (Figure 1d,e), a considerable number of chloride ions are free. Ongoing hydration of cement densifies the interface between the sensor and the cementitious material. Densification of the interface results in the presence of fine pores of nm size between the sensor and hydration products. The physical binding of chloride ions to the surface of hydration products lowers the activity of chloride ions at the sensor’s surface. The lower activity of chloride ions will be reflected in the sensor’s response. The presence of preferentially oriented crystalline phases at the interface (Section 2.1) limits the dissolution/precipitation processes at the sensor’s surface. This will be reflected in the sensor’s response. Consequently, the properties of the sensor and surrounding cementitious materials can affect the thermodynamically plausible reactions at the sensor’s surface. This is due to the limitations of electron/ion transport at the sensor surface and/or limitations caused by the resistive properties of the surrounding medium. The AgCl layer properties, different cement hydration products and their compactness at the interface between sensor and bulk matrix are the major factors that limit the sensor performance. If the morphology and microstructure of the AgCl layer, binding of chloride ions to the hydration products and the pore size distribution around the sensor are not considered, the readings of chloride sensor are difficult to interpret. Thus, a link between the inherent properties of the sensor and the relevant exposure environment has to be made. To overcome the lack of information on the performance of chloride sensor in cementitious materials, further research is needed.

## 7. Conclusions

The available techniques for determination of the free chloride content in cementitious materials are prone to several sources of errors. The potentiometric technique, using an Ag/AgCl chloride sensor, is one of the most promising methods for non-destructive in situ free chloride measurement. The deviation of the sensor’s OCP from Nernstian behaviour has been considered as the main criterion for evaluation of the sensor performance. The causes of inaccurate sensor measurement are often a concern. The available studies superficially commented on the short- /long-term stability of the chloride sensor, paying little attention to the causes of inaccuracies. As a result, the identification of the underlying sources of inaccuracies is a prerequisite for the practical application of the sensor.

A reliable and reproducible chloride sensor measurement requires knowledge of the environmental exposure condition and the inherent properties of the sensor. In this regard, the following should be considered:

1. The interfacial properties between the silver substrate and the AgCl layer, i.e., morphology of the AgCl particles and the conductivity of the layer.
2. The presence of interfering hydroxide and sulphide ions, i.e., the pore water composition.
3. The relative distribution and compactness of cement hydration products at the sensor’s surface, i.e., the occurrence of “screening” effects.

The physical properties of the AgCl layer (porosity, thickness, morphology, etc.) affect the rate of ion exchange and dissolution/precipitation processes at the sensor’s surface. Instability of Ag/AgCl chloride sensor is typically due to dissolution of the AgCl layer and/or the formation of other silver compounds. As a result, mixed potential subsequently develops at the sensor’s surface. The AgCl layer is more stable when the chloride concentration is high, while at low concentrations, products other than silver chloride form. The presence of other silver compounds obviously affects the performance of the sensor. If the aim is to determine the chloride content close to the reinforcement at the time of corrosion of reinforcement with high accuracy, the sensor’s response should be reliable in a wide range of chloride concentration, i.e., 0.045–3.22 mol/L (Table 2). As the source of chloride ions is mainly from the external environment, the time required for chloride ions to penetrate into the concrete and reach at least 0.045 mol/L at the sensor surface can be very long. Consequently, the sensor will be exposed to an alkaline medium free of chloride ions or with a very low chloride content for a relatively long period of time. In such a case, the accuracy of the chloride sensor cannot be simply judged on the thermodynamic principles, depending on the reaction kinetics at the sensor’s surface.

The microstructure of cementitious materials is the resistive component that can limit the ion transport at the sensor surface and affect the thermodynamic plausible reaction of the chloride sensor. Cementitious materials also serve as a diffusion barrier for an embedded chloride sensor, slowing down the rate of AgCl dissolution. This feature can be beneficial for the long-term performance of the chloride sensor. The dissolution of the AgCl layer can be fatal for the sensor performance. The dissolved AgCl particles can gradually diffuse into the medium, exposing the Ag substrate to direct contact with the environment. Moreover, the presence of hydroxide and/or sulphide ions in cementitious materials can interfere with the Nernstian behaviour of the sensor. The significance of different cement hydration products and their compactness around the sensor is from the subsequent influence on the activity of the chloride ions. The alteration in chloride ions activity is subsequently reflected in the sensor’s response. Therefore, recognition of the imposed limitations from these parameters is important for reliable chloride sensor measurement.

## Figures and Tables

**Table 1:** Concentration of ions in the pore solution of hydrated Portland cement paste (w/c = 0.5) after a 28-day curing period [9].

	Concentration (mmol/L)					
	Na^+^	K^+^	Ca^2+^	Cl^−^	SO_4_^2−^	Ionic strength (mmol/L)
Hydrated cement paste	88.9	74.8	4.8	1.4	0.2	178.3

**Table 2:** Different expression forms of the critical chloride content and the range of reported chloride threshold values for steel, embedded in cement-based materials.

Aggressive Species	Expressed as	Reported Chloride Threshold Value	Reference
Total chloride (including free chloride)	% by weight of binder	(1.24–3.08), (1–8.34), (0.68–0.97)	[38,39,40]
	% by weight of concrete	(0.03–0.07), (0.03–0.2), (0.06–0.2) (0.06–0.37)	[41,42,43,44]
Free chloride	% by weight of binder	(0.39–1.16), (1-4), (0.5–2)	[33,38,39]
	% by weight of concrete ^1^	0.026	[45]
	mol/L	(0.36–3.22), (0.44–0.65), (0.045–0.55)	[46,47,48]
	[Cl^−^]/[OH^−^]	(1.17–3.98), (1.7–20), (3–20)	[38,39,49]

^1^ Expression of critical free chloride content by weight of concrete is scarcely available in the literature.

**Table 3:** Gibbs free energies for the formation of species in silver–halide–water systems at 25 °C [96].

Species	Gibbs Free Energy (KJ/mol)	Species	Gibbs Free Energy (KJ/mol)
Ag^+^	77	Ag(OH)_2_^−^	−260
Cl^−^	−131	AgCl (c)	−110
Ag_2_O (c)	−11	AgCl (aq)	−54
AgO (c)	15	AgCl_2_^−^	−216
AgOH	−80	AgCl_4_^3−^	−478

(aq): aqueous phase—(c): crystalline phase.
